# How Accumulated Real Life Stress Experience and Cognitive Speed Interact on Decision-Making Processes

**DOI:** 10.3389/fnhum.2017.00302

**Published:** 2017-06-08

**Authors:** Eva Friedel, Miriam Sebold, Sören Kuitunen-Paul, Stephan Nebe, Ilya M. Veer, Ulrich S. Zimmermann, Florian Schlagenhauf, Michael N. Smolka, Michael Rapp, Henrik Walter, Andreas Heinz

**Affiliations:** ^1^Department of Psychiatry and Psychotherapy, Charité Campus Mitte (CCM), Charité-Universitätsmedizin BerlinBerlin, Germany; ^2^Biomedial Innovation Academy, Berlin Institute of HealthBerlin, Germany; ^3^Department for Social and Preventive Medicine, University of PotsdamPotsdam, Germany; ^4^Department of Psychiatry and Psychotherapy, Institute of Clinical Psychology and Psychotherapy, Technische Universität DresdenDresden, Germany; ^5^Neuroimaging Center, Technische Universität DresdenDresden, Germany; ^6^Department for Human Cognitive and Brain Sciences, Max Planck Institute LeipzigLeipzig, Germany

**Keywords:** chronic stress, model-based learning, model-free learning, decision making, cognitive speed, real-life events

## Abstract

**Rationale:** Advances in neurocomputational modeling suggest that valuation systems for goal-directed (deliberative) on one side, and habitual (automatic) decision-making on the other side may rely on distinct computational strategies for reinforcement learning, namely model-free vs. model-based learning. As a key theoretical difference, the model-based system strongly demands cognitive functions to plan actions prospectively based on an internal cognitive model of the environment, whereas valuation in the model-free system relies on rather simple learning rules from operant conditioning to retrospectively associate actions with their outcomes and is thus cognitively less demanding. Acute stress reactivity is known to impair model-based but not model-free choice behavior, with higher working memory capacity protecting the model-based system from acute stress. However, it is not clear which impact accumulated real life stress has on model-free and model-based decision systems and how this influence interacts with cognitive abilities.

**Methods:** We used a sequential decision-making task distinguishing relative contributions of both learning strategies to choice behavior, the Social Readjustment Rating Scale questionnaire to assess accumulated real life stress, and the Digit Symbol Substitution Test to test cognitive speed in 95 healthy subjects.

**Results:** Individuals reporting high stress exposure who had low cognitive speed showed reduced model-based but increased model-free behavioral control. In contrast, subjects exposed to accumulated real life stress with high cognitive speed displayed increased model-based performance but reduced model-free control.

**Conclusion:** These findings suggest that accumulated real life stress exposure can enhance reliance on cognitive speed for model-based computations, which may ultimately protect the model-based system from the detrimental influences of accumulated real life stress. The combination of accumulated real life stress exposure and slower information processing capacities, however, might favor model-free strategies. Thus, the valence and preference of either system strongly depends on stressful experiences and individual cognitive capacities.

## Introduction

Habitual responding to rewards and the pursuit of goals are key to human decision-making. Such habitual (automatic) vs. goal-directed (planned) control of behavior is associated with distinct neural systems for valuation and decision-making (Dolan and Dayan, 2013). Computational attempts to understand these behavioral systems assume that both reflect different computational strategies during reinforcement learning, namely model-based (goal-directed) vs. model-free (habitual) behavior (Daw et al., 2005, 2011; Dolan and Dayan, 2013). Therefore, there is a close association between the theoretical concepts of goal-directed and model-based behavior (Friedel et al., 2014; Gillan et al., 2015; Sjoerds et al., 2016). A key difference between the two behavioral strategies is that model-based behavior strongly demands higher cognitive functions to plan actions prospectively based on an internal model of the environment. Model-free behavior on the other hand relies on simple retrospective evaluation of cached reward values and is cognitively less demanding (Daw et al., 2005; Otto et al., 2013a; Schad et al., 2014). The conditions under which each of these systems controls behavior have been of particular interest in neuroscience and psychiatry, in part because the (im)balance between model-free and model-based behavior is believed to be a key factor in a number of psychiatric disorders. Psychiatric conditions characterized by increased model-free behavior at the cost of model-based performance are e.g., addiction, binge eating disorder and obsessive compulsive disorder (Sebold et al., 2014; Voon et al., 2015; Gillan et al., 2016; Heinz et al., 2017). Among situational factors that influence reward-based decision making, stress is a key candidate for biasing the balance of the two systems toward more habitual decision making (Schwabe and Wolf, 2009, 2012) and might thus be of relevance for the development and maintenance of these disorders.

One key factor in the arbitration between model-based and model-free behavior may be the interaction between stress and cognitive functioning, as stress is known to exert strong influences on cognition and learning (Baumeister et al., 2002; Garrett et al., 2010; Otto et al., 2013b). The magnitude and valence of this influence crucially differs depending on the operationalization (e.g., pain, social stress), the timing and duration of acute vs. chronic stress exposure (Lupien et al., 2009) and the specific cognitive function. Acute stress is known to impair goal-directed choices (Schwabe and Wolf, 2009) and executive functions underlying model-based behavior (Otto et al., 2013a). Executive cognitive functions that have been strongly associated with model-based behavior are processing speed (Schad et al., 2014) and working memory capacity (Otto et al., 2013b; Smittenaar et al., 2013; Schad et al., 2014).

Recent reports of decision-making under stress (for review see Starcke and Brand, 2012) primarily focused on effects of acute stress (Schwabe and Wolf, 2009; Otto et al., 2013b; Buckert et al., 2014). For example, it has been shown that acute stress, as indicated by a transient cortisol response of the neuroendocrine system to a laboratory stressor disrupts context-dependent memory (Schwabe et al., 2009), induces a shift from more goal-directed towards habitual strategies (Schwabe and Wolf, 2009) and impairs model-based behavior (Otto et al., 2013b). Crucially, high working memory capacity protects individuals from this disruption (Otto et al., 2013b), suggesting that stress interacts with executive functions underlying model-based control.

On a neurobiological level the mesostriatal dopamine system is prominently implicated in reinforcement learning in humans with neural signals in the ventral striatum covarying with prediction error signaling during reinforcement learning (Kurniawan et al., 2013). We have previously shown that the neural correlate of reinforcement learning in the ventral striatum is moderated by cognitive functioning and chronic (accumulated real life) stress experience (Friedel et al., 2015). This signal in the ventral striatum has also been shown to be influenced by acute stress (Robinson et al., 2013) and changes in cortisol levels during an acute stressor were correlated with increases in striatal responses during a decision-making task (Dedovic et al., 2009). On the behavioral level, stress facilitates a shift from flexible cognitive to more rigid habit memory systems (Schwabe and Wolf, 2009). On the neural level this is in line with the idea of reduced prefrontal cortex functions such as working memory and attention, promoting a switch from “thoughtful “top-down” control by the prefrontal cortex to “bottom-up” control by the amygdala and related subcortical structures” (Arnsten, 2009; Yu, 2016).

However, while negative influences from acute stress on decision-systems are well documented, little is known about how accumulated real life stress exposure affects cognitive functions underlying model-based choice. Evidence from animal studies indicates that chronically stressed rats turn towards habitual behavior (Dias-Ferreira et al., 2009) and one study suggests that this finding might be translated to human decision-making (Soares et al., 2012). A recent study reports that acute and chronic (accumulated real life) stress may interact: acute stress exposure reduce model-based behavior, but only in subjects earlier exposed to high levels of chronic (accumulated real life) stress (Radenbach et al., 2015). These findings underline the importance of chronic stress in behavioral control. However, the cognitive and computational mechanisms underlying such influences are still insufficiently understood.

Here, we used the Social Readjustment Rating Scale (Holmes and Rahe, 1967) to study how accumulated real life stress interacts with cognitive speed on model-based vs. model-free decision-making in a sample of healthy subjects. First, we explored contributions of model-based vs. model-free decision-making via statistical analysis of choices in a sequential decision-making task. Following up on statistical findings, we tested our primary hypothesis that stress and cognitive speed interact with model-based and model-free decision-making as indicated by the interaction between transition frequency (common vs. rare) and reward.

## Materials and Methods

### Subjects and Screening Instruments

A group of *N* = 95 right handed healthy adults (*N* = 16 females) with a mean age of 43.62 years (*SD* = 11.0; range: 21.4–66) and an average of 11.3 years of school education (*SD* = 1.5 years) was recruited in a longitudinal German two-center study on learning and alcohol dependence (LeAD, see www.lead-studie.de; Clinical Trials identifier NCT01744834). The reported sample of healthy subjects had been matched to a sample of alcohol dependent subjects according to their age, smoking status and gender. We excluded one subject based on implausible high stress values (SRRS = 635 > 3 *SD* of the group’s mean of 122). The study consisted of 2 days of testing, including 1 day of psychopathological assessment, neuropsychological tests, and questionnaires, and a second day involving fMRI scanning during two experimental learning tasks. Neuropsychological testing included the digit symbol substitution test (DSST, Wechsler, 1997) as a measure of cognitive speed and working memory capacity as assessed with the digit symbol backwards test (Wechsler, 1997; Aster et al., 2006) which had previously been associated with model-free and model-based control (Otto et al., 2013b). Moreover, we have assessed the German version of the verbal knowledge test (MWTB, Lehrl, 2005), which relates to crystallized IQ (Schad et al., 2014) and the trial-making-test (TMT) -A and B, which assesses executive functioning (Corrigan and Hinkeldey, 1987; Sánchez-Cubillo et al., 2009).

On the first day, written informed consent was obtained from all participants before they underwent the neuropsychological testing. On the second day, participants completed the below-described sequential decision making task (Daw et al., 2011). After completing the task, participants received monetary compensation for their participation. Ethical approval for the study was obtained in accordance with the Declaration of Helsinki from the Medical Ethics Committees of Charité–Universitätsmedizin Berlin (EA/1/157/11) and Technische Universität Dresden (EK 228072012). Not included were subjects with Axis I psychiatric disorders except nicotine dependence, alcohol abuse and specific phobia according to DSM-IV as measured with the Composite International Diagnostic Interview (CIDI, Wittchen and Pfister, 1997; Jacobi et al., 2013), subjects with DSM-IV personality disorders (SAPAS screening; Moran et al., 2003), and subjects with MRI contraindications (for further details see Sebold et al., 2016).

### Measures of Accumulated Real Life Stress and Cognitive Speed

#### Accumulated Real Life Stress

In the Social Readjustment Rating Scale (SRRS, Holmes and Rahe, 1967) participants indicated whether any of 43 potentially stressful life events occurred to them within the last 12 months. Each life event is associated with a specific amount of life change units (LCUs) based on ratings by a large sample of participants, ranging from 100 LCUs for “death of a spouse” to 11 LCUs for “minor violation of the law”. The LCUs for each of these life events were added up, providing a measure of past-year stress load for each participant, which has been proven to be a reliable indicator of overall (and lifetime) accumulated real life stress (Holmes and Rahe, 1967; Scully et al., 2000).

#### Cognitive Speed

**Figure 1:**
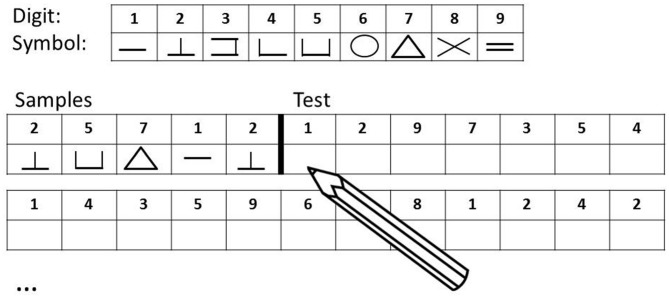
Digit Symbol Substitution Test (DSST, Wechsler, 1997): the DSST consists of a code table displaying pairs of digits and symbols, and rows of double boxes with a digit in the top box and an empty space in the bottom box. The task for the subject is to use the code table to determine the symbol associated with each digit, and to write as many symbols as possible in the empty boxes below each digit within 120 s.

Cognitive speed was assessed with the DSST (Wechsler, 1997), see Figure 1. The DSST is a neuropsychological test measuring general processing speed (Salthouse, 1992), writing speed, and short-term-memory (Laux and Lane, 1985). Subjects are provided with a code table assigning nine different abstract symbols to the digits 1–9 and are then given a table presenting a list of digits in each top row and empty boxes in each bottom row. They are then instructed to sequentially draw as many of the 133 (maximum score) corresponding symbols underneath the digits as possible in 120 s. Standardized values corrected for age according to the manual (DSST, Wechsler, 1997) resulted in scores from 2 to 19 which were used for subsequent analyses.

### Two-Step Task

**Figure 2:**
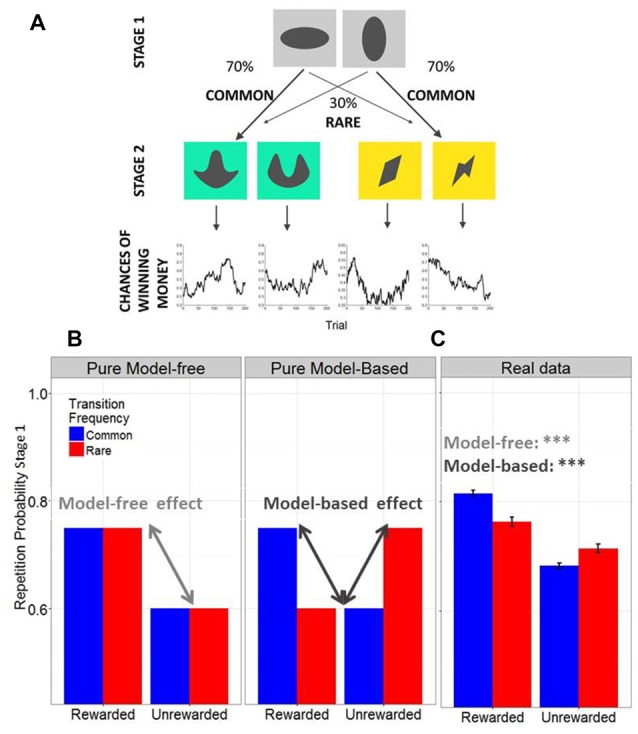
**(A)** Structure of the Two-Step Task. **(B)** Simulated data of a pure model-free vs. a pure model-based decision-maker. Model-free and model-based strategies predict distinct response patterns on the first stage. In model-free decisions, first stages should be repeated whenever the outcome of the previous trial was rewarded, whereas they should not be repeated whenever choices were unrewarded. Therefore model-free decisions predict a main effect of reward on first stage repetition of the subsequent trial. In model-based decisions, the individual takes transition frequencies into account. Thus, for instance, when a trial from the rare transition frequency ended up in reward, the individual knows that in order to obtain reward in the next trial he/she should actually switch to the opposing first stage stimuli, because this one has a higher probability of ending up at the specific second stage stimulus pair that is now associated with high probability of reward. **(C)** Across all subjects (*N* = 95), model-free and model-based scores were significantly positive (as indicated with the *** both *p* < 0.0001), suggesting that subjects showed a mixture of model-free and model-based choice strategies.

We adapted the Two-Step decision task (Daw et al., 2011, Figure 2) for MATLAB with the Psychophysics Toolbox Version 3 extension (Brainard, 1997; Pelli, 1997) in order to assess model-based vs. model-free decision making. We used a new set of colored stimuli, but the same transition structure and outcome probabilities as in the original Two-Step study (Daw et al., 2011). Participants had to choose one out of a pair of abstract grayscale stimuli leading to another colored stimulus pair for choice at stage 2 (stage 1 and 2 at Figure 2A). Instructions emphasized reward maximization. Importantly, the win probability for each of the four stage 2 stimuli varied over time according to a slow and independent random walk (chances of winning money at Figure 2A). The probability to be presented with a specific stimulus pair at stage 2 depended on the choice at stage 1 and was constant over time; there was a common (70%) and a rare (30%) transition for each stage 1 stimulus. After the experiment, one third of all rewards (with a fixed minimum of 3 EURO and maximum of 10 EURO) was additionally paid out to increase the motivation of the participants. The instructions provided detailed information about the structure of the task; specifically concerning the varying outcome probabilities at stage 2 and about the constant transition probabilities between stage 1 and 2. In addition, there were 50 practice trials prior to the main experiment. The distinction between model-based and model-free performance primarily depends on the use of the transition probability: a purely model-free learner would repeat a decision that led to a rewarded choice ignoring the transition frequencies, resulting in a main effect of reward on selection of the first stage; whereas a purely model-based learner would repeat a decision that led to a rewarded common choice but most unlikely repeat a decision that led to a rewarded rare choice, resulting in an interaction between transition frequency (common vs. rare) and reward (see Figure 2B).

### Data Analysis

The final sample consisted of 95 subjects with a mean DSST score of 10.8 (*SD* = 3.1) and a mean SRRS score of 115.7 (*SD* = 94.9). All regression analyses were conducted using linear models implemented in the stats package of the R programming language, version 3.1.2 (cran.us.r-project.org). For orthogonal contrasts (rewarded vs. unrewarded/common vs. rare), we used effect coding [−0.5 0.5]. The level of statistical significance was set to *p* < 0.05.

We were specifically interested in how model-free and model-based control were related to stress and cognitive speed. For this purpose, we calculated two individual scores, one for model-free (% rewarded common + % rewarded rare – % unrewarded common – % unrewarded rare, see Figure 2B, left plot) and one for model-based behavior (% rewarded common + % unrewarded rare – % rewarded rare – % unrewarded common, see Figure 2B, middle plot), as previously described (Sebold et al., 2014). Individual model-free and model-based scores were extracted from the raw data of the Two-Step task, where the percentage of individual first stage repetitions was calculated based on the previous trial’s outcome (rewarded vs. unrewarded) and transition frequency (common vs. rare, see Figure 2B). Model-free effects describe the individual main effect of reward, whereas individual scores for model-based control reflect the interaction between transition frequency and reward. In line with our previous research (Friedel et al., 2014; Sebold et al., 2014), we chose this analysis strategy because we aimed to extract individual model-free and model-based scores in order to subsequently predict differences in both scores from stress and cognitive speed.

**Figure 3:**
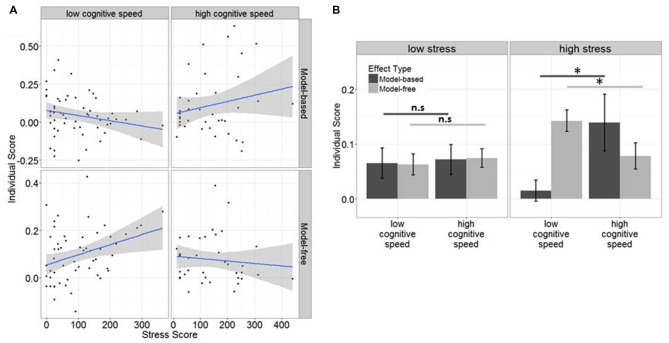
**(A)** Effects of stress and cognitive speed on model-free vs. model-based control: subjects with low cognitive speed display an increase of model-free but a decrease of model-based behavior with increasing stress exposure, whereas subjects with high cognitive speed display an increase of model-based but a decrease of model-free behavior with increasing stress exposure. **(B)** The differential association of cognitive speed with model-free and model-based control was particularly evident in subjects who had experienced high stress (significant effects indicated with *), whereas there was no such effect in subjects who had experienced low stress.

The scores (both approaching normal distribution) then served as criterion variable in two subsequent linear regressions, in which the interaction between stress and cognitive speed was tested on each of these scores. We computed median splits of DSST score (which were normally distributed) and subjects were assigned to a high or low cognitive speed group (low ≤ 11, high > 11), before entered into the regression model. SRRS scores were z-transformed before they were entered into the regression models as a continuous variable. For further *post hoc* tests and illustrative purposes (see Figure 3B), we additionally assigned subjects to a low (≤ 101) and a moderate to high (>101) stress group based on the group’s median split.

As previous research has indicated that differences in age could impact on the balance between model-free and model-based control (Eppinger et al., 2013; Sebold et al., 2016), our results could potentially have been confounded by age effects. In order to test this, we performed additional analyses, where we put age (z-scaled) as an additional nuisance regressor in the two previously described linear models.

In order to replicate previous studies, which demonstrated that subjects showed a mixture between model-free and model-based decision-making strategies in the Two-Step task, we performed one-sample *t*-tests, which tested whether each score was significantly larger than zero.

## Results

Across all subjects, model-free and model-based scores were significantly positive (both *p* < 0.0001), suggesting that subjects showed a mixture of model-free and model-based choice strategies (see Figure 2C).

In the linear model regressing cognitive speed, stress and their interaction on model-free control, no main effect was found for either stress (*p* = 0.16), nor cognitive speed (*p* = 0.30), while their interaction (*β* = 0.5, *F*_(91)_ = 2.76, *p* = 0.022) was significantly associated with model-free behavior. In fact, model-free control increased with increasing stress exposure when cognitive speed was low (see Figure 3A).

In the second linear model we again found no main effect of cognitive speed (*p* = 0.1) nor stress (*p* = 0.86) on model-based control, but an interaction between stress and cognitive speed which was negative, (*β* = −0.07, *F*_(91)_ = 2.7, *p* = 0.04), indicating that model-based behavior was reduced when stress exposure was high but cognitive speed was low (see Figure 3A).

Healthy controls reported rather low levels of accumulated real life stress, which led to a positive (left) skew of the data. When dichotomizing the SRRS score effects closely failed to reach significance (on a *p*-level of 0.05: interaction of stress and cognitive speed on model-free control, *p* = 0.08; interaction of stress and cognitive speed on model-based control, *p* = 0.09). This seems plausible, as the sample consisted of healthy control subjects, where most subjects reported a comparably little amount of accumulated real life stress. To maintain most of the variance of the SRRS predictor variables (which is reduced by performing median splits) and in line with previous research (Radenbach et al., 2015) we stuck with the above reported analyses. The assumptions for a multiple regression analysis were met.

The digit symbol backwards test (Wechsler, 1997; Aster et al., 2006), the German version of the verbal knowledge test (MWTB, Lehrl, 2005) and the TMT-A and -B (Reitan and Wolfson, 1985) did not interact with stress regarding either model-free or model-based control.

When age was added as additional covariate in our two linear models, we again found that the interaction between stress and cognitive speed was significant for model-free (*β* = 0.05, *F*_(90)_ = 2.07, *p* = 0.022) and model-based control (*β* = −0.07, *F*_(90)_ = 2.05, *p* = 0.04), suggesting that the interaction between stress and cognitive speed on model-free and model-based control was not caused by confounding age effects. Moreover, in these analyses, we found no main effect of age on model-free (*p* = 0.78) or model-based control (*p* = 0.67), suggesting that in our sample age did not impact these decision-making strategies.

Exploratory *post hoc* analyses, where we tested the influence of cognitive speed on model-free and model-based decision-making separately for low and high stress subjects revealed that cognitive speed increased model-based (*β* = 0.06, *p* = 0.025) but decreased model-free control (*β* = −0.03, *p* = 0.04) only in high stress subjects, whereas this effect was not significant in low stress subjects (model-based: *β* = 0.02, *p* = 0.4, model-free: *β* = −0.13, *p* = 0.34). Thus, differences between the influence of cognitive speed on model-free vs. model-based control were mainly driven by subjects who had experienced comparably high stress in the past year (see Figure 3B).

## Discussion

The main finding of our study is that self-reported life stress exposure during the past 12 months interacts with cognitive processing speed on human decision-making: in individuals with lower cognitive speed, accumulated real life stress was associated with reduced model-based performance and a shift towards model-free choice behavior. High levels of cognitive speed, on the other hand, seemed to protect individuals from such influences, as these were associated with an increased reliance on the model-based system, especially after high accumulated real life stress exposure.

Acute stress is known to induce a distinct decline of model-based choices, while leaving the model-free system unaffected (Otto et al., 2013b). High working memory capacity, a measure closely related to cognitive flexibility and processing speed, was recently shown to protect model-based choices from such deteriorating influences (Otto et al., 2013b). This suggests that the relative transition from model-based towards model-free decision-systems (Schwabe and Wolf, 2009; Otto et al., 2013b) observed after acute, laboratory-induced stress might be due to taxing of executive functions underlying model-based decision-making. The present findings replicate findings that high processing speed is related to model-based decision-making (Schad et al., 2014) and complement on previously reported interaction effects between acute stress reactivity and cognitive abilities (Otto et al., 2013b) by pointing to the influence of chronic, long-term real-life stress. With respect to the interaction of acute and chronic stress experience on model-based decision making, Radenbach et al. (2015) have shown that acute stress results in a decrease of model based performance only when chronic (accumulated real life) stress exposure was high. In addition to and complementing on this finding, we show that the association of real-life stress with model-based decision making is also modulated by cognitive speed.

An important question arising from our findings is which mechanisms underlie the association between long-term stress, cognitive speed and the balance between model-based vs. model-free control. Empirical evidence on the differential influence of acute vs. long term stress on cognitive functioning is in part controversial. Acute stress is known to exert a negative influence on memory. Schwabe and Wolf (2009) report that acutely stressed individuals become insensitive to the devaluation of a particular outcome accompanied by a significant decrease in explicit knowledge of action-outcome contingencies. For enduring past stress experience time-dependent effects on working memory processing, emotional memory and brain function in general have been reported (for a recent overview see Yu, 2016). Chronic enduring stress exposure in humans led to a compensatory upregulation of prefrontal functioning whereas acute stress and the quick influence of cortisol in combination with noradrenaline led to an increase of subcortical and a decrease of prefrontal functioning (Hermans et al., 2011, 2014). Findings from animal research demonstrate that chronically stressed rats become insensitive to devaluation of outcomes (Graham et al., 2010), together with atrophy of the medial prefrontal cortex and hypertrophy of the putamen (Dias-Ferreira et al., 2009) indicating a negative influence of long term stress exposure on cognitive processing.

Looking from an evolutionary perspective, Decker et al. (2016) suggest that the recruitment of model-based valuation systems relies on a critical cognitive component, which is associated with the gradual maturation of goal-directed behavior. Whereas a model-free strategy was apparent in choice behavior across all age groups, a model-based strategy was absent in children, emerged in adolescents and matured in adults. This observation suggests that cognitive resources like processing speed may be decisive in coping with the influences of long-term real-life stress experience depending on brain maturation and age. In this context, the complexity and uncertainty associated with accumulated real life stress may pose difficulties and expose limits for the development of fully rational decision strategies and favor computationally less demanding model-free decision-making strategies (Daw et al., 2005; Otto et al., 2013b).

One important aspect of the mechanisms associated with an overreliance on model-free strategies is their association with the development and maintenance of various psychiatric disorders, such as addiction, obsessive compulsive disorder and binge eating disorder (Everitt and Robbins, 2005; Sebold et al., 2014; Voon et al., 2015). Gillan et al. (2016) used a transdiagnostic approach and applied the Two-Step task to about 2000 healthy individuals assessing a broad variety of symptoms (assessed via a self-report questionnaire containing 209 items), which have been associated with different psychiatric diagnostic categories (e.g., alcohol addiction, schizotypy, depression and social anxiety). Independent of the diagnostic category, the authors report a strong association of compulsive spectrum behavior with a decrease in model-based performance in the Two-Step task. Decision-making tendencies might thus be linked to certain trait markers that interact with the vulnerability for the development of compulsive spectrum disorders. Also, past stress exposure increases the risk for the development of psychiatric disorders, such as major depressive disorder and schizophrenia (Murgatroyd and Spengler, 2011a,b, 2012) and might further add up to an increased vulnerability with respect to its interaction with individual cognitive capacities and decision making strategies.

An important limitation to our findings is that they are correlational and from a cross-sectional design, hence no causal conclusions should be drawn and assumptions on the employment of cognitive abilities according to environmental circumstances are theoretical. However, the different directional associations are interesting to disentangle. The importance of our findings derives from the fact that we complement previous findings on acute stress (Otto et al., 2013b) with chronic (accumulated real life stress) exposure, strengthening the importance of both (past) environmental as well as cognitive variables in understanding human decision making. Coping abilities associated with high processing speed might be able to enhance model-based decision-making in response to stressful experiences. Further, especially longitudinal research is needed to disentangle effects of acute vs. chronic (accumulated real life) stress exposure and cognitive abilities on choice systems in different mental disorders, and to closely parse the cognitive and computational processes underlying the interaction of processing performance speed and past stress experiences in model-based decision-making.

To account for accumulated real life stress effects, we used the weighted sum of (positive and negative) life events reported by the subjects in the past 12 months with the SRRS. The generalizability of our results is thus limited, as we could not account for the interindividual differences in the experience of accumulated real life stress, which can potentially be influenced by an (im)balance of personal traits, resources and the demands placed upon an individual by social and occupational situations. However, an interesting feature of the SRRS is that it spans a broad range of events and their estimated potential to elicit readjustment processes, including events that are usually related to positive affect (such as pregnancy, marriage and outstanding personal achievement). Crucially these events may be regarded positively by some and negatively by others, depending on the context of change (such as changes in residence, changing to a different line of work, major changes in responsibility at work). Moreover the SRRS includes negative events (such as death of spouse, death of a close family member). All of these events have been rated and evaluated according to their need for social readjustment on large independent samples (Holmes and Rahe, 1967; Scully et al., 2000).

Due to a lack of statistical power we could not assess the influence of sex on decision making and its interaction with accumulated real life stress. However, this aspect is worth mentioning, as there is recent evidence that sex differences are important modulators of stress-related reward sensitivity and decision making (for a recent overview see Yu, 2016). It was found that stress led to greater reward collection and faster decisions in males but less reward collection and slower decisions in females (Lighthall et al., 2012). One study showed that mild psychological stress resulted in a significant decrease in reward-related responses in the medial prefrontal cortex without affecting ventral striatal responses in women (Ossewaarde et al., 2011).

We did not assess physiological measures of stress. A biological correlate of chronic stress such as hair cortisol, which could give some important information on the hypothalamic–pituitary–adrenal (HPA) axis in the months before assessment, should be considered in future studies. Given the effects of cortisol on cognition, this could have an effect on model-based/model-free learning as well (Otto et al., 2013b; Radenbach et al., 2015).

Altogether, our findings suggest that the cognitive abilities and processes underlying model-based decision-making may not be fixed (Schad et al., 2014), but are rather flexibly employed according to environmental circumstances. While model-based computations build on executive resources and processing speed, especially when past experiences have already demanded flexible adaptation to ever changing environments (such as stress induced through a high need for social readjustment), other settings seem to foster model-free processes. A preference for model- free strategies might prevail after high stress exposure especially when experience has shown that the possibility of a fast flexible adaptation has been insufficient (e.g., due to low processing speed).

## Author Contributions

AH conceived the study. AH, HW, MR, MNS, USZ and FS designed the study protocol; EF, MS, SK-P, SN collected the data; EF, MS and IMV analyzed the data; EF, MS, SK-P, SN, IMV, FS, USZ, MNS, MR, HW and AH interpreted the data and wrote the manuscript. All authors viewed the final version of the manuscript prior to submission and are accountable for all aspects of the work. EF and MS contributed equally.

## Conflict of Interest Statement

The authors declare that the research was conducted in the absence of any commercial or financial relationships that could be construed as a potential conflict of interest.
